# USA300 Methicillin-resistant *Staphylococcus aureus *in Cuba

**DOI:** 10.1186/2047-2994-1-2

**Published:** 2012-01-26

**Authors:** Joost Hopman, Gilda Toraño Peraza, Fidel Espinosa, Corné H Klaassen, Dayneris Menéndez Velázquez, Jacques F Meis, Andreas Voss

**Affiliations:** 1 Radboud University Nijmegen Medical Centre, Nijmegen, The Netherlands; 2 Pedro Kourí Institute, Department of Bacteriology-Mycology, Havana, Cuba; 3 Hermanos Ameijeiras Hospital, Department of Microbiology, Havana, Cuba; 4 Canisius Wilhelmina Hospital, Department of Medical Microbiology and Infectious Diseases, Nijmegen, The Netherlands

**Keywords:** MRSA, Cuba, Caribbean, Infection control, Hospital-associated-infection, Low-resource setting

## Abstract

**Background:**

Methicillin-resistant *Staphylococcus aureus *is an increasing problem in the Caribbean. We investigated the molecular epidemiology of MRSA isolates on Cuba.

**Findings:**

The predominant clone was of the spa type t149, followed by community-associated MRSA USA300.

**Conclusions:**

We report the first molecular typing results of MRSA isolates from Cuba.

## Findings

In this study we aim to investigate the molecular epidemiology of methicillin-resistant *Staphylococcus aureus (*MRSA) clinical isolates from 4 major hospitals in Cuba. MRSA is an increasing problem in the Americas and the Caribbean including Cuba[1-4]. Although epidemiological data on MRSA in Cuba is available, so far no molecular typing has been performed[1,3]. Percentages of nosocomial *S. aureus *isolates resistant to methicillin ranges between 6% in Cuba to 85% in Peru[1]. Recently, MRSA isolates are emerging as significant pathogens in the community [3,5]. In the USA, the most prevalent CA- MRSA clone is USA300 (CC8, spa type t008, PVL+). In settings with a high prevalence of MRSA, MRSA is an important cause of nosocomial infections [6]. High MRSA prevalence rates have major consequences for infection control policies in hospitals. Microbiological surveillance is an important tool to evaluate this local infection control policy and to assess which microorganisms are causative pathogens in these settings. Surveillance per se probably results in a decrease of nosocomial infections as is shown by the International Nosocomial Infection Control Consortium (INICC)[7]. INICC and the World Health Organization's global program of "clean care, is safer care" advocate cost-effectiveness of microbiological surveillance in low-resource settings. Despite the fact that device-associated (DA-) and other healthcare-associated infection (HAI) rates are much higher in low and middle income countries, most studies on MRSA and HAIs have been conducted in high resource countries [7-9]. A prospective cohort study in two adult intensive care units of Cuban university hospitals showed a DA-HAI overall rate of 30.6 (95% CI 27.8-33.5) per 1000 ICU-days. DA-HAI rates for Ventilator Associated Pneumonia (VAP), Central line-associated bloodstream infection (CLA-BSI) and catheter-associated urinary tract infections (CAUTI) were used to calculate the overall DA-HAI rate. CLA-BSI rates ranged from 1.9 to 2.3 per 1000 central line-days[10]. As *Staphylococcus spp*. is an important pathogen of CLA-BSI's, the existence of MRSA in Cuba could result in increased treatment failures. This could later pose an even higher burden of HAI's on the Cuban health care system. A system with great emphasis on disease prevention and primary care rather than on medical supplies and technologies [11]

During a 3 months period in 2008 all clinical isolates suspected to be MRSA were collected prospectively at three major Cuban hospitals and the national reference centre for infectious diseases, the Pedro Kouri Institute Havana, The Pediatric Hospital "William Soler" Havana, The General Hospital "Hermanos Ameijeiras" Havana and the General hospital "Manuel Ascunse" Camaguey. Registration of the origins of isolate cultures as well as standard microbiological testing, including oxacillin susceptibility testing (by CLSI) was performed in Cuba. Only clinical relevant isolates were included. No further clinical data was available. The origins of isolate cultures were wounds, bronchial/tracheal aspirations, blood, skin, abdominal drainage and chest tissue biopsy in 13, 8, 7, 5, 5, 1 and 1 isolates, respectively. Further molecular testing was done in the Netherlands, including detection of Panton-Valentine leukocidin (PVL), *mecA*, the Sa442 element as well as typing by and spa-typing.

Of the 68 suspected Staphylococcus spp. isolates that were sent to the Netherlands, 40 were *mecA *positive and confirmed to be MRSA. Thus routinely performed phenotypical identification of MRSA isolates had a high false positive rate 41%. Spa typing identified 5 different spa-types. In decreasing frequency we found spa-type t149 (n = 24), t008 (n = 8), t037 (n = 6), and one each of t4088 (closely related to t149) and t2029 (closely related to t037). All eight t008 isolates but none of the other spa types were PVL positive. Multi locus sequence typing (MLST) was performed on t008 isolates and isolates proved to be CC8 positive.

CA-MRSA USA300 was first described in the USA where it has become endemic. Subsequently, this strain has been spread to Latin America [3]. Given Cuba's isolation and the USA's embargo on travel, we were surprised to find that USA300 was the second most common strain found in this study. It highlights the widespread localization of this strain over the Americas.

Surgical-site infections (SSI) are the leading health-care-associated infection hospital-wide in the developing world[7]. In our study 35% of the cultures originated from wounds obtained after surgery. This demonstrates the importance of microbiological testing and infection control measures aiming to prevent SSI's. Preventing MRSA positive SSI's is probably cost-beneficial, as the treatment of these infections is performed with second and third line antibiotic regimens. Those antibiotics are often difficult to obtain in low and middle-income countries, are more expensive and as such pressing on pre-existing limited financial resources.

In conclusion we report the first molecular typing results of MRSA isolates from Cuba. The predominant clone was the spa-type 149, followed by community associated MRSA USA300.

## Competing interests

The authors declare that they have no competing interests.

## Authors' contributions

JH performed the analysis and drafted the manuscript. GP collected the isolates and clinical data and coordinated the study in Cuba, FE collected the isolates and clinical data and coordinated the study in Cuba, CK carried out the molecular genetic studies and helped to draft the manuscript. DV collected the isolates and clinical data and coordinated the study in Cuba JM conceived the study, and participated in its design and coordination and helped to draft the manuscript AV conceived the study, and participated in its design and coordination and helped to draft the manuscript. All authors read and approved the final manuscript.
